# Foliar Application of Iron Nanoparticles Improves Chinese Cabbage Growth

**DOI:** 10.3390/plants14223509

**Published:** 2025-11-17

**Authors:** Miaomiao He, Jialu Yu, Yuzhen Wei, Fahad Munir, Fasih Ullah Haider, Liqun Cai

**Affiliations:** 1 College of Resources and Environmental Sciences, Gansu Agricultural University, Lanzhou 730070, China; 1073323020464@st.gsau.edu.cn (M.H.); 107332202431@st.gsau.edu.cn (J.Y.); feinibuke19851026@163.com (Y.W.); 2 Key Laboratory of Dry Land Crop Science, Gansu Agricultural University, Lanzhou 730070, China; fasihullahhaider281@gmail.com; 3 Postharvest Research and Training Centre, Institute of Horticultural Sciences, University of Agriculture, Faisalabad 38040, Pakistan; fahadmunir5544@gmail.com

**Keywords:** nano−zero−valent iron, nanofertilizer, foliar application, photosynthetic fluorescence, antioxidant defense

## Abstract

Iron deficiency limits plant growth and is usually addressed with iron fertilizers. Iron−based nanomaterials (nZVI, α−FeOOH, α−Fe_2_O_3_, γ−Fe_2_O_3_, and Fe_3_O_4_) show promise as novel alternatives, but the effects of sulfide nano−zero−valent iron (S−nZVI) on crops remain little studied. Thus, this study aimed to synthesize a novel iron−based nanomaterial, S−nZVI, using a one−step method, and to evaluate the effects of S−nZVI and nZVI at concentrations ranging from 5 to 100 mg L^−^^1^ on the physiological and photosynthetic characteristics of Chinese cabbage (*Brassica rapa* L.). In the study, foliar application of iron nanoparticles increased leaf area, biomass, and photosynthesis, with 50 mg L^−^^1^ the most efficient concentration (S−nZVI > nZVI). Moreover, the photosynthetic rate of the leaves increased significantly (>200%), and carbohydrate accumulation also increased significantly. Additionally, S−nZVI treatment increased leaf iron content by 5.8−fold compared to the control group, likely by enhancing the activity of antioxidant enzymes. However, the 100 mg L^−^^1^ S−nZVI treatment significantly inhibited these physiological and biochemical indicators. Overall, the foliar S−nZVI (50 mg L^−^^1^) enhanced Chinese cabbage growth by alleviating iron deficiency, boosting antioxidant activity, and reducing oxidative stress; further field trials are needed to verify its effectiveness and cost−efficiency.

## 1. Introduction

Iron deficiency is a common condition worldwide. According to statistics, approximately 40% of the world’s soil lacks bio−effective iron, particularly in calcareous soils [1]. A survey report on soil trace elements across 31 provinces in China reveals that iron deficiency is more prevalent in the northern provinces than in the southern provinces. Gansu and Shaanxi have the most serious iron deficiency in the north [2]. Hence, to minimize iron deficiency, iron deficiency in plants is often improved by applying a large amount of traditional iron fertilizer in agricultural production. However, this agricultural model is quietly evolving into an environmental crisis: excessive fertilizer drives freshwater eutrophication, algal blooms squeeze the living space of aquatic animals and plants, and toxins are more likely to spread along the food chain, threatening the health of residents [3,4]. Moreover, the availability of iron in soil usually varies with soil conditions (e.g., pH). In alkaline, calcareous soil, iron readily forms insoluble compounds, such as hydroxides, leading to iron−deficiency chlorosis in plants [5]. Therefore, developing efficient, environmentally friendly, and high−utilization iron fertilizers is crucial to mitigating iron deficiency and enhancing agricultural productivity.

In recent years, nanotechnology has garnered interest in agriculture for its ability to enhance nutrient delivery and mitigate environmental impacts [6]. Their small particle size and large surface area facilitate enhanced nutrient uptake by plant roots and leaves. In general, iron−based nanomaterials (NMs) made from nano−zero−valent iron (nZVI), nano−hematite (α−Fe_2_O_3_), nano−goethite (α−FeOOH), nano−magnetite (Fe_3_O_4_), and nano−ferrite (γ−Fe_2_O_3_) can serve as novel iron−based fertilizers. Compared with traditional fertilizers, it offers many advantages, including higher crop yields, improved nutrient uptake, and reduced environmental pollution [4]. Iron nanoparticles possess unique properties, including a high surface−to−volume ratio, biocompatibility, and catalytic activity, making them suitable for agriculture, environmental management, and medicine [7,8]. Numerous studies have demonstrated that iron nanoparticles can effectively enhance crop growth and development. For example, research by Jiangbing et al. [9] demonstrated that treating lettuce (*Lactuca sativa* L.) with low concentrations of nano−Fe_3_O_4_ particles significantly enhanced its biomass and net photosynthetic rate, resulting in increases of 15.15% and 3.37%, respectively. Additionally, 50 mg L^−1^ of Fe_3_O_4_ NMs can increase chlorophyll content by 26.9% and mitigate stress effects [10].

Existing studies have shown that adding 0.5 g L^−1^ nano−zero−valent iron can promote *Arabidopsis* root elongation, with a promotion efficiency of 150% to 200%. Yoon et al. [11,12] reported that nZVI can significantly improve biomass, photosynthetic efficiency, and iron content in the body of Pseudomonas. Previous studies have also reported the negative effects of nano−zero−valent iron. For example, high doses of nano−zero−valent iron can induce excessive reactive oxygen species, cause DNA damage, and reduce the mitotic index [12]. However, organisms also have a highly effective iron homeostasis mechanism that can buffer the effects of exogenous iron within a certain range. Therefore, before introducing nano−zero−valent iron into farmland, it is urgent to systematically clarify the dose−effect relationship to resolve the dispute between risk and benefit. Moreover, zero−valent iron is readily oxidized and prone to agglomeration. How to achieve ‘sustained release + low toxicity and high efficiency’ on the leaf surface is rarely evaluated.

Hence, in this study, ‘sulfur’ was used as a switch to construct an Fe−S layer−controlled−release system, and its dose−effect inflection point was verified. Utilized Chinese cabbage as a representative model to comprehensively evaluate the effects of two iron−based nanomaterials (S−nZVI NMs and nZVI NMs) on the physiological processes of Chinese cabbage under different concentrations of foliar spraying, aiming to select iron−based NMs as the optimal application concentration of iron fertilizer and determine the optimal type of iron−based NMs. It was hypothesized that applying S−nZVI would stimulate these physiological processes, fostering enhanced growth and increased resilience to adversity. The outcomes of this study aim to provide a scientific framework for the effective, practical implementation of iron−based nanomaterials as fertilizers in crop production.

## 2. Results

### 2.1. Characterization of Two Kinds of Iron−Based Nanomaterials

**Figure 1:**
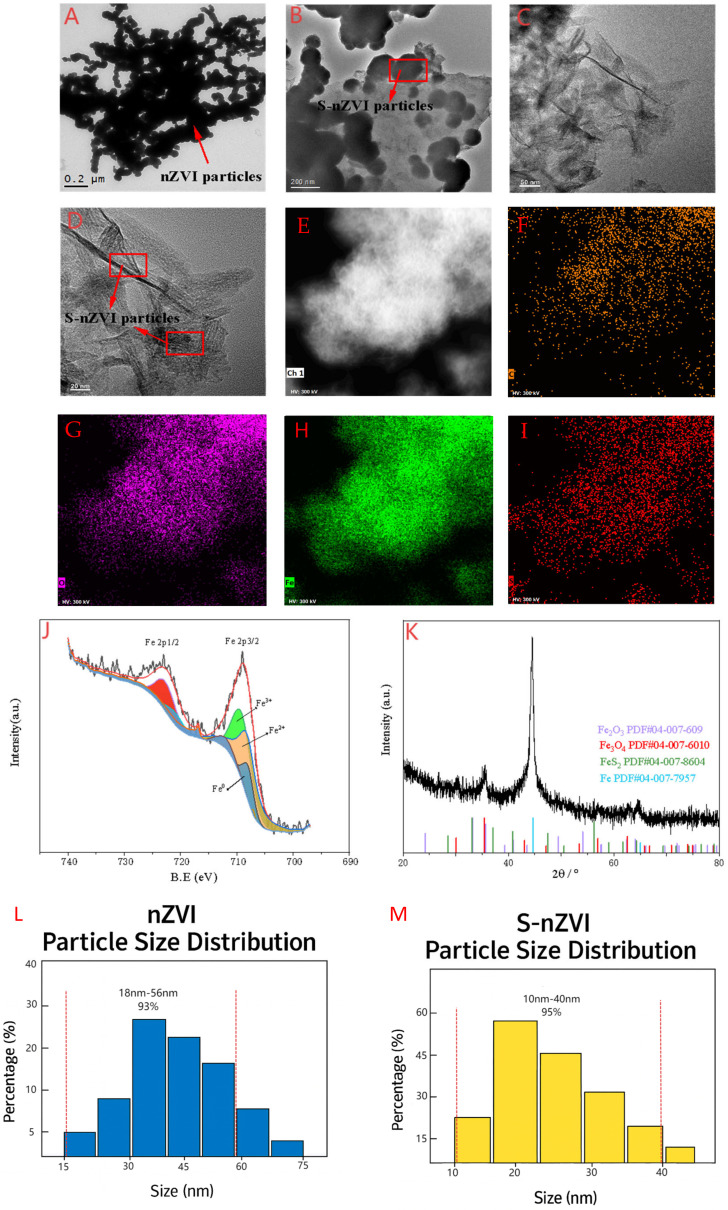
Transmission electron microscopy (TEM) images of (**A**) nanoscale zero−valent iron (nZVI) and (**B**–**D**) sulfide−modified nanoscale zero−valent iron (S−nZVI) at different resolutions; (**E**) energy−dispersive X−ray spectroscopy (EDS) spectrum of S−nZVI; (**F**–**I**) elemental mapping of carbon (C), oxygen (O), sulfur (S), and iron (Fe) in S−nZVI; (**J**) X−ray photoelectron spectroscopy (XPS) spectrum and (**K**) X−ray diffraction (XRD) pattern of S−nZVI; (**L**) Statistics of nZVI particle size distribution; (**M**) S−nZVI particle size distribution statistics. Note: The red dotted lines in (**L**) and (**M**) represent the range of particle size.

The transmission electron microscope (TEM) of the nZVI (nanoscale zero−valent iron) and S−nZVI is shown in Figure 1. The morphological structure of nZVI (Figure 1A) shows that the material exhibits a core–shell structure at a resolution of 200 nm, and the whole is spherical particles, which are almost all aggregated into clusters. In contrast, the synthesized S−nZVI at 200 nm (Figure 1B) is enveloped in a sulfide layer, and the particles are relatively dispersed. After further enlarging the material resolution to 50 nm (Figure 1C) and 20 nm (Figure 1D), it can be more clearly observed that the agglomeration effect of nZVI particles after vulcanization treatment is significantly alleviated, and the vulcanization layer is uniformly coated on the surface of nZVI particles. This phenomenon indicates that the sulfide layer successfully coats nZVI particles. Figure 1E shows the EDS analysis results of S−nZVI. The elements on the surface of S−nZVI, in addition to C and O (Figure 1F,G), also enriched a large amount of S and Fe (Figure 1H,I), indicating that the sulfide modification successfully coated zero−valent iron, reduced its agglomeration, and improved its dispersion. This result was consistent with TEM analysis.

The X−ray photoelectron spectroscopy (XPS) results for S−nZVI, shown in Figure 1J, focus on the Fe 2p energy spectrum. A characteristic peak of Fe^0^ appears at a binding energy of 709.6 eV, confirming that zero−valent iron remains the dominant phase in the composite after sulfide modification. Additionally, characteristic peaks of Fe^2+^ (Fe 2p3/2) and Fe^3+^ (Fe 2p3/2) are observed at 709.12 eV and 711.68 eV, respectively, indicating the presence of oxidized iron in the material. Based on these results, it can be inferred that sulfide elements are successfully incorporated onto the iron surface, forming a sulfide coating that effectively protects the core structure of zero−valent iron and inhibits further oxidation. This conclusion was consistent with the XRD data, confirming the successful modification of nZVI via vulcanization treatment. The characterization analysis indicates that S−nZVI enhances material dispersion and reduces particle aggregation. Sulfidation significantly improves both its efficacy and stability. The excellent performance of S−nZVI can be attributed to the unique Fe−S layer structure, which enhances its antioxidant capacity by wrapping the zero−valent iron core with a protective sulfide layer. The X−ray diffraction (XRD) analysis of S−nZVI, as shown in Figure 1K, reveals characteristic peaks at 35.5° and 56.5°, corresponding to Fe_2_O_3_ and Fe_3_O_4_, respectively. A strong diffraction peak at 44.6° corresponds to the characteristic peak of Fe^0^, which aligns with the XRD results of the S−nZVI material. These findings indicate that the sulfide modification successfully coats the surface of nZVI.

Figure 1L shows the statistics of the nZVI particle size distribution. The data indicate that approximately 93% of the iron nanoparticles have particle sizes ranging from 15 to 56 nm. S−nZVI particle size distribution statistics (Figure 1M). The data indicate that approximately 95% of the iron nanoparticles have particle sizes ranging from 10 to 40 nm. The agglomeration effect of S−nZVI is lower than that of nZVI; the surface charge of S−nZVI is significantly higher than that of nZVI (Table 1). Avellan et al. [13] demonstrated that NMs with a negative surface charge exhibit higher bioavailability, and the higher the surface charge of NMs, the greater their bioavailability. Therefore, the bioavailability of S−nZVI is higher than that of nZVI.

### 2.2. Effects of Different Iron−Based Nanomaterials on the Growth Indices of Chinese Cabbage

The influence of varying nZVI and S−nZVI concentrations on Chinese cabbage growth parameters is summarized in Table 2. Compared to the control group (T0), several growth indicators showed statistically significant differences (*p* < 0.05). The 50 mg L^−1^ S−nZVI treatment yielded the most pronounced growth enhancement (*p* < 0.01), with increases of 6.51 cm^2^ in leaf area, 2.80 cm in plant height, 3.83 g in fresh weight, and 0.40 g in dry weight. Under the optimal growth−promoting concentration, the effect of foliar spraying S−nZVI on the DW and FW of the aboveground part of Chinese cabbage was significantly higher than that of nZVI. Conversely, treatments at 5 mg L^−1^ and 100 mg L^−1^ did not produce significant changes in leaf area, fresh weight, or dry weight relative to the control.

### 2.3. Effects of Different Iron−Based Nanomaterials on Enzyme Activity and Content

**Figure 2:**
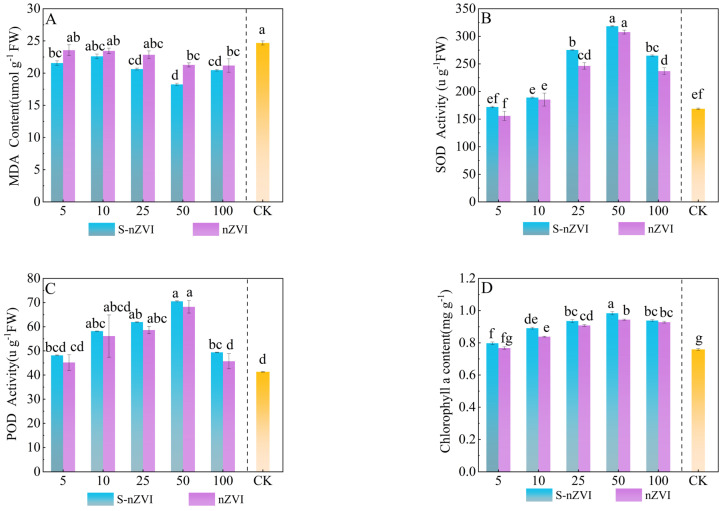

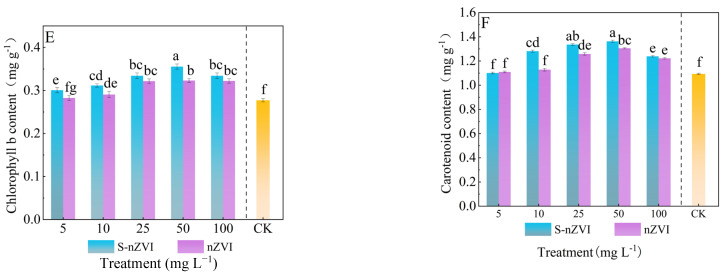
The antioxidant capacity of Chinese cabbage under control, nZVI, and S−nZVI treatments. The 10−day−old seedlings were treated, and the leaves of 45−day−old seedlings were collected to determine malondialdehyde (MDA) content (**A**), superoxide dismutase (SOD) activity (**B**), peroxidase (POD) activity (**C**), and three photosynthetic pigments: chlorophyll a content (**D**), chlorophyll b content (**E**), and carotenoid content (**F**). The value is the mean ± standard deviation (n = 6), and the bar chart shows the standard deviation. Columns with different letter markers indicated significant differences between treatments (*p* < 0.05, Duncan test).

The results of Chinese cabbage enzyme activity (Figure 2A–C) showed that foliar spraying with two types of iron−based NMs promoted enzyme activity in a dose−dependent manner. The optimal growth−promoting concentration for Chinese cabbage was 50 mg L^−1^. Under the optimal growth−promoting concentration, the effect of foliar spraying S−nZVI on the increase in MDA content in Chinese cabbage was significantly higher than that of nZVI. MDA levels initially decreased and then increased as the nZVI and S−nZVI concentrations increased, with the lowest MDA content observed at 50 mg L^−1^, resulting in decreases of 11.60% and 25.69% compared with the control group (T0). At 100 mg L^−1^, MDA content reached the peak of 21.03 and 20.45 µg g^−1^. POD and SOD activities exhibited similar patterns: both enzymes showed significant increases when leaves were sprayed with 25 mg L^−1^ and 50 mg L^−1^ S−nZVI. With POD rising by 49.88% and 70.76%, respectively, compared to T0. At 50 mg L^−1^, POD activity declined. SOD activity also peaked at 50 mg L^−1^, reaching 88.62% above control levels. It is worth noting that there was no significant difference in POD and SOD activity at the optimal concentration of the two iron−based nanomaterials sprayed on the leaves; however, a significant difference was observed at the high concentration of 100 mg L^−1^.

### 2.4. Effects of Different Iron−Based Nanomaterials on Photosynthetic Pigments and Parameters

Figure 2D–F shows the effects of foliar application of S−nZVI and nZVI on the photosynthetic pigment content of Chinese cabbage. At the dose of 50 mg L^−1^, sulfur−modified nano−zero−valent iron (S−nZVI) treatment increased the contents of chlorophyll a, chlorophyll b and carotenoids to 0.98,0.35 and 1.36 mg g^−1^, respectively, which were significantly increased by 30.67%, 29.62% and 15.60% compared with the control (T0) (*p* < 0.05), and the increase was better than that of ordinary nano−zero−valent iron (nZVI) at the same concentration. The dose−dependent response peaked at 50 mg L^−1^. When the concentration increased to 100 mg L^−1^, pigment synthesis was significantly inhibited, indicating that high−dose S−nZVI may reduce photosynthetic pigment accumulation through oxidative stress or stomatal limitation.

The results of Table 3 showed that foliar application of iron−based NMs could significantly increase the net photosynthetic rate, transpiration rate, stomatal conductance, and intercellular carbon dioxide concentration of Chinese cabbage. Under the treatment of 50 mg L^−1^ S−nZVI, the net photosynthetic rate and intercellular carbon dioxide concentration reached the maximum, which increased by 205.8% and 158.2%, respectively. Under the treatment of 50 mg L^−1^ nZVI, the net photosynthetic rate increased by 115.3%, and the intercellular carbon dioxide concentration increased by 112.2%. The two iron−based NMs treatment groups also significantly increased stomatal conductance and transpiration rate in Chinese cabbage.

Based on further analysis of the initial fluorescence (F0) changes in the leaves of the two iron−based NMs, Table 4 shows that the initial fluorescence of each treated leaf tends to decrease and then increase with the growth and development of cabbage. The initial fluorescence (F0) of 100 mg L^−1^ is the lowest. The initial fluorescence value of the blades in each treatment group ranges from 0.13 to 0.25, indicating no significant difference from the T0 value. As shown in Table 4, with increasing nZV and S−nZVI concentrations, the Fv/Fm ratio initially increased, then decreased. In T4 processing, it reaches the highest value of 0.69 and 0.75, which were 16.95% and 27.11% higher than T0. Compared with T0, the actual photochemical efficiency of photosystem II in the S−nZVI treatment groups increased by 18.52%, 27.78%, 25.93%, 66.67%, and 37.04%, in the respective groups.

### 2.5. Effects of Different Iron−Based Nanomaterials on Trace Elements

**Figure 3:**
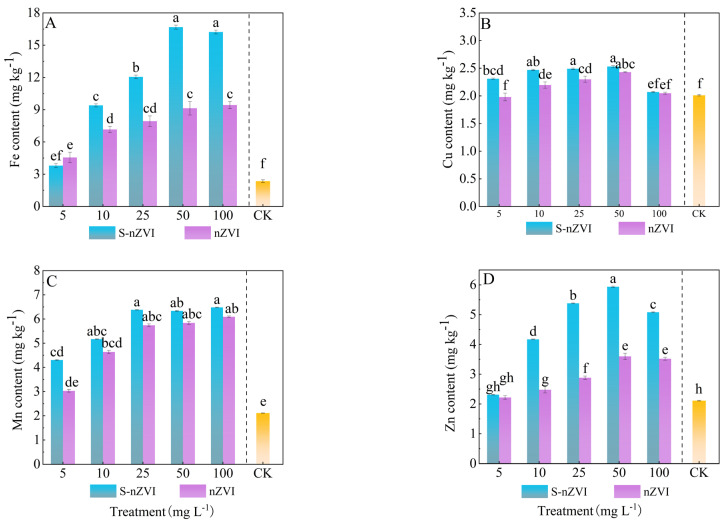
Trace elements in Chinese cabbage under control, nZVI, and S−nZVI treatments. The seedlings of 10 days old were treated, and the leaves of 45 days old were collected to determine the content of Fe (**A**), Mn (**B**), Cu (**C**), and Zn (**D**). The value is mean ± standard deviation (n = 6), and the bar chart represents the standard deviation. Columns with different letter markers indicated significant differences between treatments (*p* < 0.05, Duncan test).

The results in Figure 3 showed that the two iron−based NMs significantly increased Fe and Zn content in Chinese cabbage leaves. The Mn content in leaves increased by 123.07% and 146.42% under nZVI and S−nZVI treatments, respectively. Compared to the control, Fe content significantly increased (*p* < 0.05) by factors of 1.7, 3.3, 4.2, 5.8, and 5.7 times higher under treatments of 5, 10, 25, 50, and 100 mg L^−1^ S−nZVI, respectively (Figure 3A). The 50 mg L^−1^ treatment notably enhanced Fe accumulation, with no significant difference observed between the 50 and 100 mg L^−1^ groups. Conversely, Cu levels 100 mg L^−1^ S−nZVI did not differ significantly from the control (Figure 3B).

### 2.6. Effects of Different Concentrations of S−nZVI on Starch and Soluble Sugar Content

**Figure 4:**
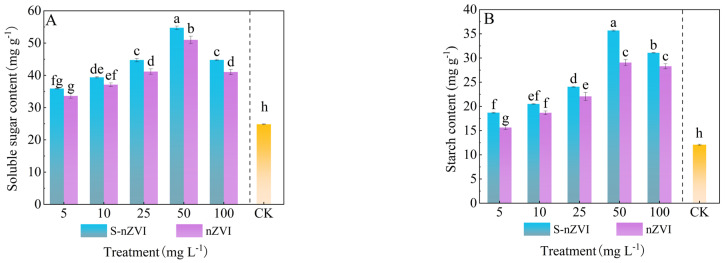
Photosynthetic products of Chinese cabbage under control, nZVI, and S−nZVI treatments. The 10−day−old seedlings were treated, and the leaves of 45−day−old seedlings were collected to determine starch content (**A**) and soluble sugar content (**B**). The value is mean ± standard deviation (n = 6), and the bar chart represents the standard deviation. Columns with different letter markers indicated significant differences between treatments (*p* < 0.05, Duncan test).

The impact of foliar−applied two kinds of iron−based NMs on the accumulation of soluble sugars and starch in Chinese cabbage leaves is illustrated in Figure 4. Both starch and soluble sugar contents increased significantly with rising S−nZVI and nZVI concentrations, peaking at 50 mg L^−1^ with values of 35.67, 29.41 mg g^−1^ and 54.74, 51.09 mg g^−1^, respectively, representing increases of 195.29%, 135.28% and 120.48%, 104.36% compared to the control. Although levels declined to 100 mg L^−1^, they remained significantly higher than those in the control. These results indicate that while excessive S−nZVI and nZVI concentrations may reduce the stimulatory effect, overall, foliar application enhances carbohydrate accumulation in the leaves.

### 2.7. PCA and Correlation Analysis

**Figure 5:**
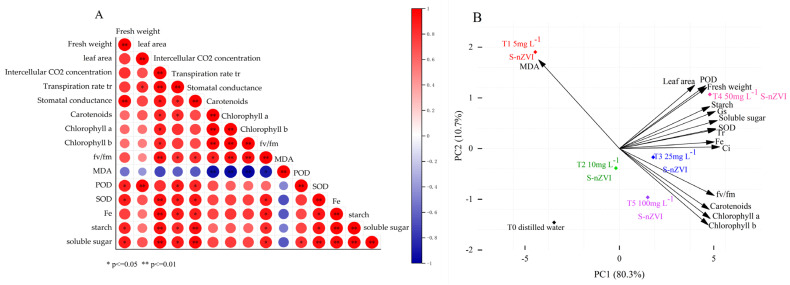
(**A**) showed the correlation analysis between different indicators (growth indicators, nutritional indicators, photosynthetic indicators, and physiological indicators); (**B**) The results of principal component analysis (PCA) showed the comprehensive effects of different S−nZVI treatments on the physiological and photosynthetic parameters of Chinese cabbage leaves. Note: *, **, in the figure indicate *p* ≤ 0.05, *p* ≤ 0.01, respectively.

Figure 5A reflects the correlation between different indicators (growth indicators, nutritional indicators, photosynthetic indicators, and physiological indicators). Iron is a key trace element that regulates the photosynthesis and respiration of plants. It can be observed that there is a significant positive correlation between iron content and fresh weight, leaf area, evaporation rate, Fv/Fm, starch, and soluble sugar. There was a substantial correlation between iron content and evaporation rate, Fv/Fm, starch, soluble sugar, chlorophyll a, and chlorophyll b. These parameters may be related to the fact that FeNPs enhance the utilization rate and conversion efficiency of energy, accelerate electron transfer rates, and improve Rubisco activity, thereby increasing photosynthetic rate and enhancing cabbage growth. Judging from the correlation in Figure 5A, there was an obvious negative correlation between the MDA content of the leaf and the iron content, evaporation rate, Fv/Fm, chlorophyll a, chlorophyll b, and soluble sugar, which showed that with the increase in S−nZVI concentration, which was conducive to the growth of biomass and the enhancement of photosynthesis, the MDA content could be effectively reduced and the oxidative damage in the plant.

A principal component analysis (PCA) was performed on 15 physiological and photosynthetic parameters of Chinese cabbage leaves under different treatments. The results are shown in Figure 5B. Principal component 1 (PC1) explained 80.3% of the total variance, while principal component 2 (PC2) explained 10.7%. Together, these two components explained 90.98% of the total variance, indicating that they can effectively represent the 15 parameters. The scores for T0, 5 mg L^−1^, 10 mg L^−1^, 25 mg L^−1^, 50 mg L^−1^, and 100 mg L^−1^ treatments in PC1 were −3.46, −4.45, −0.18, 1.79, 4.79, and 1.50, respectively. The scores for PC2 were −1.46, 1.90, −0.38, −0.17, 1.07, and −0.96, respectively. PC1 was negatively correlated with MDA and positively correlated with other parameters. PC2 was positively correlated with fresh weight, leaf area, Fe content, intercellular CO_2_ concentration, stomatal conductance, transpiration rate, SOD, POD, MDA, starch, and soluble sugars. Meanwhile, it was negatively correlated with other parameters. These two components comprehensively reflect the effects of different S−nZVI treatments on the growth and photosynthesis of Chinese cabbage. The 50 mg L^−1^ S−nZVI treatment showed the most positive impact on photosynthetic fluorescence and growth.

## 3. Discussion

### 3.1. Effects of Different Iron−Based Nanomaterials on Growth and Photosynthetic Characteristics of Chinese Cabbage

Iron deficiency often leads to chlorosis and tissue necrosis, resulting in stunted growth and reduced crop yields. Compared with conventional chelated iron fertilizers, iron−based nanomaterials offer a promising solution to iron deficiency [14]. Foliar application is efficient because nutrients can penetrate leaf nanoscale pores more readily [15]. This study demonstrated that foliar spraying of iron−based NMs at 50 mg L^−1^ increased the shoot dry weight of Chinese cabbage by 6% compared with nZVI (*p* < 0.05). The leaf area was 3% higher than that of nZVI. This may be because sulfur modification can slow the burst release of nZVI by forming an Fe−S passivation layer. At the same time, it provides sulfur nutrition and activates the glutathione−ascorbic acid cycle, thereby reducing ROS, promoting carbon assimilation, and promoting crop growth [16]. However, in another study, higher concentrations may inhibit growth due to nanoparticle aggregation on root surfaces, pore blockage, and the formation of insoluble Fe^3+^ oxide layers that restrict water and nutrient uptake [17]. Similar findings by Du et al. [18] showed that silica nanoparticles (SiO_2_−NPs) at a concentration of 100 mg L^−1^ promoted rice root and leaf growth by entering through stomata. However, at 3000 mg L^−1^, accumulation around stomata impaired their function, inhibiting water absorption and growth. These observations indicate that nanoparticle effects on plants are concentration−dependent, typically showing a low−dose stimulation and high−dose inhibition pattern.

Iron plays a crucial role in chlorophyll biosynthesis, and its deficiency impairs chlorophyll production, thereby reducing photosynthetic efficiency [19]. A study by Sepehrzadegan and Alizadeh et al. [20] reported that treatment with iron nanoparticles (Fe−NPs) enhanced the carotenoid, chlorophyll a, and chlorophyll b contents of triticale compared to conventional iron fertilizers. Li Huifang et al. [21] found that nanoparticle properties can influence CO_2_ uptake by affecting stomatal aperture. However, numerous studies have highlighted the phytotoxicity of metal oxide nanoparticles, often linked to the excessive release of harmful metal ions or reactive oxygen species (ROS), as noted by Labille et al. [22]. These findings suggest that iron nanoparticles can have both beneficial and detrimental effects on plants by altering their physiological and phenotypic traits. In this study, foliar application of 50 mg L^−1^ iron−based nanomaterials significantly increased chlorophyll content in Chinese cabbage leaves, potentially by enhancing enzymatic activity, such as δ−aminolevulinic acid synthase (δ−ALA), involved in chlorophyll synthesis [23]. Conversely, concentrations exceeding 100 mg L^−1^ led to declines in chlorophyll content and photosynthetic rates. Supporting this, Jafari et al. [24] observed reduced chlorophyll levels in motherwort at 1000 mg L^−1^ S−nZVI, likely due to nutrient competition at high nanoparticle concentrations, which aligns with the inhibitory effects seen in this study.

Chlorophyll fluorescence parameters in plants can indicate the absorption and transformation of photosynthetic products, as well as changes in plant physiological state. These changes not only affect the dynamic balance of the carbon cycle but also play an essential role in plant growth and development [25,26,27]. In this study, the 50 mg L^−1^ iron−based NMs treatment, applied via leaf surface spraying, shows the greatest effect, increasing Fv/Fm and ΦPSII (the actual photochemical efficiency of photosystem II) by 25% compared to the control (T0). The results indicate that foliar application of S−nZVI can enhance photo−synthetic activity, improve the light energy conversion efficiency of PSII in the leaves, and promote photosynthesis in cabbage leaves. This may be because S−nZVI provides a Fe−S cluster structure, similar to the [2Fe−2S] or [4Fe−4S] clusters in ferredoxin, which serves as the electron transporter in the photosystem II (PSII) of plant photosynthesis, directly affecting the rate of plant photosynthesis [28]. Under the treatment of 50 mg L^−1^ iron−based nanomaterials, the content of other nutrients (such as Fe, Mn, Zn, etc.) related to photosynthesis in the plant also increased, indicating that the leaf spraying of iron−based nanomaterial S−nZVI can not only improve the iron content in the plant but also increase the absorption and utilization of other nutrients by plants. It is worth noting that the Fe content in leaves under S−nZVI treatment was 1.3–1.4 times that of nZVI (5.8 times that of the control at 50 mg L^−1^), indicating that the sulfide layer promoted leaf absorption and transport. This finding was consistent with the conclusion of Desouza et al. [6], who reported that foliar spraying of iron−based nanomaterials significantly increased the mineral nutrient content in purslane. Under the treatment of 100 mg L^−1^ S−nZVI, the Fe content decreased, possibly due to the increased active oxygen content in the cabbage induced by excessive S−nZVI, which inhibited photosynthesis and led to Fe loss [29].

Iron is an essential micronutrient that regulates photosynthesis and respiration in plants [30]. Leaf iron content closely correlates with net photosynthetic rate and intercellular CO_2_ concentration, as stomatal pores mediate gas exchange and transpiration, thereby influencing photosynthetic efficiency [31]. Research has demonstrated that iron−based nanomaterials can enhance plant growth; for example, nano−iron treatments increased chlorophyll levels and photosynthesis in soybean (*Glycine max* L.) and white oak (*Quercus alba* L.) [32,33]. Additionally, nanoscale zero−valent iron (nZVI) can function like a natural semiconductor, capturing solar energy and converting it into electrical energy to promote growth [34]. However, high concentrations of nZVI (above 200 mg L^−1^) are toxic, inhibiting growth in species such as cattail [35]. These findings suggest that iron nanoparticles can have both beneficial and adverse effects on plants, influencing their physiological and phenotypic traits. In the present study, foliar application of iron−based nanomaterials at 50 mg L^−1^ significantly enhanced net photosynthesis, stomatal conductance, and intercellular CO_2_ concentration in Chinese cabbage. This improvement may stem from increased energy conversion efficiency, accelerated electron transport, and enhanced Rubisco enzyme activity, boosting photosynthetic performance and plant growth. Notably, the stimulatory effects diminished at 100 mg L^−1^, consistent with the typical low−dose stimulation and high−dose inhibition pattern reported in previous studies [32,33,34].

### 3.2. Effects of Different Iron−Based Nanomaterials on the Physiological and Antioxidant Properties of Chinese Cabbage

Soluble sugar and starch content are key indicators of plant carbon transformation and accumulation [36]. The significant increase in soluble sugar and starch content under 50 mg L^−1^ and 100 mg L^−1^ S−nZVI treatments indicates that nano−sulfidized iron participates in plant carbon metabolism, regulating the accumulation of starch and soluble sugars [37]. This further confirms that S−nZVI benefits carbon metabolite accumulation and promotes crop growth.

SOD can protect cells against the damage caused by reactive oxygen free radicals and repair damaged cells promptly. POD is a kind of redox enzyme produced by plants, which can catalyze the oxidation of hydrogen peroxide, phenol oxides, and hydrocarbons, while eliminating toxic substances such as hydrogen peroxide, phenols, and amines [38]. The content of MDA can reflect the degree to which plants are affected by adversity. It is often used as an indicator of lipid peroxidation to measure the degree of peroxidation of cell membrane lipids, plant aging, and the intensity of the response to adversity [39]. This study showed that the MDA content of S−nZVI was 12% lower than that of nZVI at 50 mg L^−1^, and the activities of SOD and POD were 10% and 15% higher, respectively, indicating that vulcanization modification reduced oxidative stress. This finding was consistent with the research results of Wang [40] and others, which showed that nano−iron can stimulate the activity of H_2_O_2_, SOD, and POD, thereby enhancing the photosynthesis and respiration of plants.

However, the 100 mg·L^−1^ S−nZVI treatment will have a specific inhibitory effect on cabbage growth. It was speculated that a higher concentration of S−nZVI may induce specific oxidative stress in plants, leading to increased ROS, cell damage, and elevated MDA content [41]. At the same time, it leads to decreased POD and SOD activity during the later growth phase. The MDA content in the 50 mg L^−1^ S−nZVI treatment was lower than that in other treatments, indicating that at 50 mg L^−1^, cabbage shows strong resistance, the least oxidation damage, and can effectively cope with environmental stimuli and pressure, resulting in good growth. Through pot experiments, this study investigates the mechanism by which foliar spraying of iron−based nanomaterials as a trace element fertilizer affects Chinese cabbage. Although some progress has been made in crop cultivation and in the agricultural application of iron−based nanomaterials, several scientific issues remain to be explored. In response to the unresolved problems, the following aspects need to be carried out in the future: (1) In the field experiment, the parallel treatment of nZVI and S−nZVI should be added to verify whether the advantages of sulfidation modification are still established under different soil pH, texture, and climate conditions. (2) Since 100 mg L^−1^ failed to cover the potential toxicity threshold of nZVI and S−nZVI, the concentration gradient of 200–500 mg L^−1^ should be added to define its dose−effect relationship accurately. At the same time, the synergistic or antagonistic effects of nitrogen, phosphorus, and other nutrients were systematically evaluated to optimize the formula of the compound fertilizer. (3) There was very little research on the migration/transformation/accumulation of sulfideized nanoparticles in the soil, the impact on soil ecosystems, and the biological effects on terrestrial plants, etc., which need further study. (4) Another limitation of this study is the absence of a conventional Fe–EDTA fertilizer control, which restricts direct comparison between S−nZVI and standard Fe fertilizers. Future work should incorporate Fe–EDTA as a reference treatment to quantitatively evaluate the relative efficiency, uptake pathways, and agronomic benefits of S−nZVI under both controlled and field conditions. (5) In addition, the present study did not directly confirm whether the applied nanoparticles adhered to or penetrated the leaf surface. Future studies should incorporate high−resolution imaging techniques, such as scanning electron microscopy coupled with energy−dispersive X−ray spectroscopy (SEM–EDS) or confocal laser scanning microscopy, to visualize nanoparticle localization and transcuticular movement. These approaches will help clarify whether S−nZVI primarily acts through surface adsorption or internalization, thereby improving the mechanistic understanding of its foliar uptake and transport. (6) Furthermore, molecular−level analyses are needed to elucidate the underlying mechanisms by which S−nZVI enhances photosynthesis and antioxidant defense. Future research should employ transcriptomic and metabolomic approaches to identify key regulatory genes, metabolic pathways, and signaling networks underlying Fe–S nanoparticle−mediated physiological responses. Integrating omics data with physiological observations will provide deeper insight into how S−nZVI modulates plant metabolism and improves stress resilience.

## 4. Materials and Methods

### 4.1. Preparation of Experimental Materials and Sulfide Nano Zero−Valent Iron

The Chinese cabbage variety used in this experiment was Hualiang 836, which was purchased from Shandong Hualiang Seeds Industry Co., Ltd., Weifang, China, with a germination rate of 85% and a growth cycle of 30–45 days. The soil used for the experiment was collected from farmland in Ma Zichuan, Dingxi City. The soil was typical yellow loam, with a uniform texture, the measured soil electrical conductivity was 0.37 dS m^−1^, an average bulk density of 1.26 g cm^−3^ in the 0–30 cm soil layer, pH of 8.39, organic matter of 10.71 g kg^−1^, total nitrogen of 0.71 g kg^−1^, total phosphorus of 1.59 g kg^−1^, available phosphorus of 27.32 mg kg^−1^, and available potassium of 151.66 mg kg^−1^. The nZVI material used in this experiment was provided by Gansu Gushuo Nano Agricultural Science and Technology Co., Ltd, Baiyin District, Baiyin, China. Based on the research of Yang Siming et al. [42], we further optimized the synthesis of sulfide nano−zero−valent iron; this experiment employs a one−step synthesis to prepare sulfide nano−zero−valent iron. NaBH_4_ (CAS 16940−66−2, purity ≥ 98.0% (AR/CP grade)) and Na_2_S_2_O_4_ (CAS 7775−14−6, purity 88%) were dissolved in 100 mL of deoxygenated water. Under constant stirring (400 rpm) and a nitrogen flow, the solution was gradually added to 400 mL of deoxygenated water containing 0.72 g of FeCl_3_·6H_2_O (CAS 10025−77−1, purity ≥ 99.0% (HG/T 3474−2014; Specification for Chemical Reagent−Iron(III) Chloride Hexahydrate (Ferric Chloride)). China Petroleum & Chemical Industry Press: Beijing, China, 2014). The mixture was stirred for 30 min to ensure sufficient reaction, then filtered under vacuum to obtain S−nZVI. In this experiment, the S/Fe ratio was controlled at 0.75.

### 4.2. Characterization of nZVI and S−nZVI and Preparation of Their Suspensions

About 50 mg of FeNPs and S−FeNPs were placed on a copper mesh and observed by FEI TALOS F200S transmission electron microscopy (Helios 5 CX, Thermo Fisher Scientific, Brno, Czech Republic), X−ray photoelectron spectroscopy (XPS Thermo Fisher Nexsa G2, Thermo Fisher Scientific, Waltham, Massachusetts, USA), and X−ray diffraction analysis (X’PertProMPD, Malvern Panalytical, Almelo, Netherlands). Image analysis and EDS were used to determine the morphology and elemental composition of S−FeNPs. The preparation of the nZVI and S−nZVI foliar spray solution involved adding 10 milligrams of nZVI and S−nZVI to 100 mL of deionized water. An ultrasonic cleaning machine (BRANSON 5800, Guangzhou, China) was used for 30 min to form a stable suspension with a concentration of 100 mg L^−1^. This solution was diluted with deionized water to obtain concentrations of 5, 10, 25, 50, and 100 mg L^−1^ for foliar spraying.

### 4.3. Experimental Design

The experiment included eleven treatments: a control (T0), where foliar spraying was conducted with deionized water, and five treatment groups with varying concentrations of nZVI and S−nZVI: T1 (5 mg L^−1^), T2 (10 mg L^−1^), T3 (25 mg L^−1^), T4 (50 mg L^−1^), and T5 (100 mg L^−1^). Each treatment was replicated six times. The experiment was conducted in pots, and foliar spraying was carried out 10 days after sowing Chinese cabbage seeds. Sprays were applied to both sides of the leaves every 10 days, totaling three sprays. Each spray delivered 0.5 mL of liquid per plant, applied using a handheld micro−sprayer equipped with a 0.3 mm cone nozzle at a pressure of 0.2 MPa. The sprayer produced ~60 μm droplets (DV_0.5_); the whole spray covered both leaf surfaces to runoff level. Aluminum foil was placed on the soil surface during spraying to prevent contamination and runoff onto the leaves. The foliar application occurred between 8:00 and 9:00 AM, and the leaves remained moist for 12 h after spraying. Sampling and analysis were performed 45 days after sowing.

### 4.4. Analytical Methods

#### 4.4.1. Plant Morphological Index Measurement for Chinese Cabbage

Plant height was measured using a Vernier caliper. Leaf area was determined using a leaf morphometer (TPYX−A, Zhejiang Jouneng Instrument Co., Ltd., Jinhua, China), with six replications for each treatment. For biomass assessment, the fresh weight of six plants per treatment was recorded. The plants were blanched in a 105 °C oven for 15 min and then dried at 80 °C until a constant weight was achieved. The dry weight was then recorded.

#### 4.4.2. Measurement of Photosynthetic Pigments and Fluorescence Parameters

Chlorophyll a, chlorophyll b, and carotenoid content were measured using acetone. Leaves were placed in the same position as the cabbage to remove the middle veins, then cut into pieces and mixed well. Weigh 0.2 g of the freshly cut sample and add it to 10 mL of acetone extract. Soak the sample in the dark for two days, shaking once every 12 h. After centrifugation, the supernatant was measured at 645 nm, 663 nm, and 470 nm using a UV−2102C spectrophotometer, with 80% acetone as the blank. The concentration of chlorophyll a, b, and carotenoids (mg L^−1^) was calculated according to the OD value of each wavelength, and the pigment content (mg g^−1^ fresh weight) was calculated according to the obtained concentration [43].

$$\mathrm{Chlorophyll}\;a\;\mathrm{concentration}=13.95\times OD\left(663\;nm\right)-6.88\times OD\left(645\;nm\right)$$

$$\mathrm{Chlorophyll}\;b\;\mathrm{concentration}=24.96\times OD\left(645\;nm\right)-7.32\times OD\left(663\;nm\right)$$

$$\mathrm{Carotenoid}\;\mathrm{concentration}=\left(1000\right.\times OD\left(470\;nm\right)-2.05\times c\left(chlorophyll\;a\right)-14.8\times c\left(chlorophyll\;b\right)/245$$

Moreover, photosynthetic fluorescence parameters were measured using a chlorophyll fluorescence meter (PAM−2000, Heinz Walz GmbH, Effeltrich, Germany), including initial fluorescence (F_0_), maximum fluorescence (Fm), Fv/Fm (the maximum photochemical efficiency of PSII), and the actual photochemical efficiency of PSII.

#### 4.4.3. Gas Exchange Parameters for Chinese Cabbage Leaves

The determination of leaf net photosynthetic rate (Pn), transpiration rate (Tr), and stomatal conductance (Gs) of Chinese cabbage leaves was measured by a photosynthetic apparatus (GFS−3000, Walz, Germany). The measurement time was from 9:00 to 12:00 AM. After the photosynthetic apparatus was preheated for 30 min, the measurement parameters were set, the zero value was adjusted, and the ‘CO_2_ Abs value’ was observed to be stable. One fully expanded leaf from each treated Chinese cabbage was clamped with tweezers and placed in the instrument leaf chamber. After the ‘photosynthetic rate A value ‘was stable, the ‘Store MP’ button was clicked to store the data, and the next leaf was replaced 5–10 times.

#### 4.4.4. Measurement of Nutrient Element in Chinese Cabbage Leaves

Leaf tissues (0.20 g DW, 100−mesh) were digested in 5 mL 65% HNO_3_ (CAS:7697−37−2, TraceMetal grade, TraceMetal grade, CNW Technologies GmbH, Düsseldorf, Germany) and 2 mL 30% H_2_O_2_ (CAS:7722−84−1, Suprapur, Merck KGaA, Darmstadt, Germany) within a MARS 6 microwave system (CEM, USA; 10 min ramp to 120 °C, hold 5 min; 10 min ramp to 180 °C, hold 20 min). After cooling, digests were made up to 50 mL with 18.2 MΩ·cm ultrapure water (Milli−Q IQ7000). Total Fe, Mn, Zn, and Cu were quantified by ICP−5000 (Focused Photonics Co., Ltd., Hangzhou, China) at 238.204, 257.610, 206.200, and 324.752 nm, respectively; Fe LOD was 0.3 µg L^−1^. Calibration (0–5 mg L^−1^, Spex CertiPrep, Metuchen, New Jersey, USA) yielded R^2^ ≥ 0.9995. Accuracy was verified with certified reference material GBW10014 (Fe 69 ± 4 mg kg^−1^; recovery 96–104%). Blanks < LOD, RSD < 3% (n = 6). The Method follows Zhao and McGrath (1994) [44].

#### 4.4.5. Measurement of Enzyme Activity and Content

Using the M0102B and M0105B assay kits provided by Suzhou Michy Biomedical Technology Co., Ltd., Suzhou Industrial Park, Suzhou, China. The activity of SOD (units g^−1^ FW) [45] and POD (units g^−1^ FW) [46] was determined by the ultraviolet absorption method. Malondialdehyde (MDA) content was determined using the TBA method. Weigh 0.1 g of different treated leaf tissues, grind and extract on ice in 1.8 mL of PBS, and centrifuge, and the supernatant is the extract. Each treatment was repeated 6 times. Take 0.2 mL of supernatant, 0.55 mL of PBS, and 0.75 mL of TBA. Shake well, then place in a boiling water bath for 10 min. After centrifugation at 3000 rpm for 15 min, the supernatant was used to measure the OD at 532 nm, 600 nm, and 450 nm, and the MDA content was calculated.

#### 4.4.6. Measurement of Starch and Soluble Sugar Content

A 0.5 g sample of fresh plant leaves was weighed, soaked in a 10 mL solution of 80% ethanol, and then soaked in 80 °C water for 30 min. The sample was centrifuged at 3000 rpm for 10 min. The supernatant was added with activated carbon and decolorized at 80 °C for 30 min. The absorbance value of the mixture of the filtrate and anthrone was determined by a spectrophotometer at 620 nm. Then create the standard curve: use a standard glucose solution of known concentration to plot it, and calculate the soluble sugar concentration based on the sample’s absorbance. The precipitate after soluble sugar extraction was boiled with 2 mL of distilled water for 15 min, cooled to room temperature, and then added to 2 mL of perchloric acid (HClO_4_) solution. The mixture was stirred for 15 min to dissolve the starch, and then centrifuged at 4000 rpm for 10 min. The supernatant was collected in a 50 mL volumetric flask, and the above steps were repeated 3 times. The absorbance of the collected supernatant was measured at 620 nm. Finally, the standard curve was created by plotting absorbance values from the standard starch solution of known concentration, and the starch concentration was calculated from the sample absorbance [47].

### 4.5. Data Analysis

Each pot (one plant) was treated as an experimental unit. Six independent replicates (n = 6) were applied per treatment. Data normality was examined using the Shapiro–Wilk test (*p* > 0.05), and homogeneity of variances was verified by Levene’s test (*p* > 0.05). One−way ANOVA followed by Duncan’s multiple−range test was performed to detect differences among treatments at a significance level of α = 0.05. All analyses were conducted using SPSS 26.0 (IBM, Armonk, New York, NY, USA), and figures were prepared with Origin 2021 (OriginLab, Northampton, MA, USA).

## 5. Conclusions

This study confirmed that sulfur−modified nano−zero−valent iron (S−nZVI) exhibits higher colloidal stability and leaf absorption efficiency due to a surface Fe−S protective layer, thereby simultaneously improving photosynthetic electron transport (Fv/Fm increased by 27%) and antioxidant defense (MDA decreased by 26%). The dose dependence was evident, and 50 mg L^−1^ was found to be the optimal concentration for foliar spraying. At this time, the dry weight increased by 6% compared with nZVI, the iron content increased by 5.8 times, and trace elements such as Mn and Zn showed synergistic increases. The results verified the potential of S−nZVI as a high−efficiency, low−dose iron nanofertilizer for typical alkaline yellow soil (pH 8.4). They provided a ‘low input−high utilization’ technology prototype for iron−deficiency vegetable production. However, there are still two limitations in the study: (1) At present, only based on the potted microcosm, the long−term interaction of soil−microorganism−nanoparticles has not been elucidated; (2) Lack of 200–500 mg L^−1^ high dose toxicity threshold and nitrogen, phosphorus, and potassium combined application effect data. In the future, it is necessary to carry out (i) field experiments in multiple ecological zones, (ii) the valence and distribution of iron in the leaf−root−soil system should be tracked using synchrotron radiation or TEM−EDS, and (iii) safety assessment of non−target microorganisms and food chains to promote S−nZVI from laboratory to commercial green iron fertilizer.

## Figures and Tables

**Table 1:** Particle size and Zeta potential of S−nZVI and nZVI.

NMs	Particle Size	Zeta Potential
S−nZVI	31.0 ± 17.7	−2.34 ± 0.17
nZVI	44.4 ± 22.3	−0.61 ± 0.11

**Table 2:** Effects of foliar treatment with nZVI and S−nZVI on growth metrics of Chinese cabbage.

Iron−Based Nanomaterials	Treatment	Leaf Area (cm^2^)	Plant Height (cm)	Number of Blades	Fresh Weight of Overground (g)	Dry Weight of Overground (g)
S−nZVI	T0	27.04 ± 0.01 g	7.74 ± 0.48 d	4.51 ± 0.09 f	1.47 ± 0.01 f	0.14 ± 0.02 c
	T1	28.50 ± 0.11 e	9.89 ± 0.08 ab	5.27 ± 0.08 e	1.81 ± 0.11 def	0.15 ± 0.01 c
	T2	31.13 ± 0.03 c	10.51 ± 0.09 ab	7.35 ± 0.18 cd	1.97 ± 0.14 def	0.34 ± 0.03 abc
	T3	32.34 ± 0.04 b	10.27 ± 0.13 ab	7.92 ± 0.15 abc	2.99 ± 0.37 bc	0.39 ± 0.06 ab
	T4	33.56 ± 0.02 a	10.55 ± 0.15 a	8.44 ± 0.14 a	5.30 ± 0.09 a	0.55 ± 0.02 a
	T5	27.62 ± 0.05 fg	10.46 ± 0.18 ab	7.92 ± 0.15 abc	2.47 ± 0.18 cd	0.29 ± 0.02 bc
nZVI	T0	27.04 ± 0.01 g	7.74 ± 0.48 d	4.51 ± 0.09 f	1.47 ± 0.08 f	0.14 ± 0.02 c
	T1	27.72 ± 0.11 f	7.93 ± 0.05 d	4.78 ± 0.05 ef	1.54 ± 0.03 f	0.14 ± 0.02 c
	T2	29.83 ± 0.15 d	8.52 ± 0.08 cd	7.04 ± 0.19 d	1.63 ± 0.02 ef	0.28 ± 0.11 bc
	T3	31.12 ± 0.32 c	9.41 ± 0.09 bc	7.33 ± 0.07 cd	2.43 ± 0.07 cd	0.36 ± 0.01 b
	T4	32.67 ± 0.08 b	10.27 ± 0.11 ab	8.03 ± 0.09 ab	3.57 ± 0.11 b	0.40 ± 0.05 b
	T5	27.30 ± 0.23 fg	10.43 ± 0.13 ab	7.65 ± 0.12 bcd	2.31 ± 0.06 cde	0.27 ± 0.01 bc

Note: The growth of Chinese cabbage under control, nZVI, and S−nZVI treatments. The 10−day−old seedlings were treated, and the leaves of the 45−day−old seedlings were collected to measure growth parameters (leaf area, plant height, plant fresh weight, and plant dry weight). FW; fresh weight, DW; plant dry weight). The mean value is ± SD (n = 6). Columns with different letter connections indicated that there was a significant difference between treatments at the *p* < 0.05 (Duncan’s multiple range test) level.

**Table 3:** Effects of foliar nZVI and S−nZVI application on photosynthetic parameters in Chinese cabbage.

Iron−Based Nanomaterials	Treatment	Net Photosynthetic Rate (μmol m^−2^ s^−1^)	Intercellular CO_2_ Concentration (μmol mol^−1^)	Transpiration Rate (mmol m^−2^ s^−1^)	Stomatal Conductance (mmol m^−2^ s^−1^)
S−nZVI	T0	6.78 ± 0.16 f	147.43 ± 5.50 e	1.28 ± 0.14 e	50.16 ± 0.34 e
	T1	7.58 ± 0.07 f	282.65 ± 14.38 c	3.33 ± 0.23 bc	53.90 ± 0.17 e
	T2	12.64 ± 0.06 e	340.10 ± 9.98 ab	3.86 ± 0.37 b	80.24 ± 0.19 d
	T3	16.98 ± 0.11 cd	382.03 ± 6.11 a	3 ± 0.26 bc	90.04 ± 0.22 cd
	T4	20.73 ± 0.21 a	380.71 ± 16.75 a	5.97 ± 0.15 a	156.45 ± 0.09 a
	T5	17.80 ± 0.31 bc	345.18 ± 13.47 ab	6.35 ± 0.19 a	97.60 ± 0.11 c
nZVI	T0	6.78 ± 0.16 f	147.42 ± 5.49 e	1.28 ± 0.14 e	50.16 ± 0.34 e
	T1	7.22 ± 0.26 f	154.18 ± 14.30 e	1.63 ± 0.094 de	52.79 ± 2.47 e
	T2	11.34 ± 0.45 e	227.22 ± 8.94 d	3.16 ± 0.14 bc	77.97 ± 4.05 d
	T3	15.42 ± 0.80 d	268.85 ± 5.14 cd	3.41 ± 0.12 bc	89.99 ± 2.25 cd
	T4	17.31 ± 0.77 bc	312.88 ± 6.43 bc	3.68 ± 0.231 b	132.93 ± 7.63 b
	T5	16.94 ± 0.50 cd	308.07 ± 5.39 bc	2.55 ± 0.29 cd	44.47 ± 3.34 e

Note: Photosynthesis of Chinese cabbage under control, nZVI, and S−nZVI treatments. The 10−day−old seedlings were treated, and the leaves of 45−day−old seedlings were collected to measure photosynthetic rate (net photosynthetic rate, intercellular CO_2_ concentration, transpiration rate, and stomatal conductance). The mean value is ±SD (n = 6). Columns with different letter connections indicated that there was a significant difference between treatments at the *p* < 0.05 (Duncan’s multiple range test) level.

**Table 4:** Effects of varying S−nZVI concentrations on fluorescence parameters in Chinese cabbage.

Iron−Based Nanomaterials	Treatment	Minimal Fluorescence F0	Maximum Fluorescence Fm	Fv/Fm PSII Maximum Photoelectrochemical Quantum Yield	Actual Photochemical Efficiency of Photosystem II
S−nZVI	T0	0.20 ± 0.01 cd	0.49 ± 0.01 h	0.59 ± 0.01 f	0.54 ± 0.01 h
	T1	0.21 ± 0.01 bc	0.58 ± 0.01 ef	0.61 ± 0.02 ef	0.64 ± 0.01 f
	T2	0.25 ± 0.02 a	0.71 ± 0.02 b	0.64 ± 0.01 ef	0.69 ± 0.02 de
	T3	0.19 ± 0.01 cd	0.68 ± 0.01 bc	0.69 ± 0.01 bcd	0.68 ± 0.02 de
	T4	0.18 ± 0.034 d	0.76 ± 0.01 a	0.75 ± 0.01 a	0.9 ± 0.01 a
	T5	0.15 ± 0.01 e	0.59 ± 0.02 ef	0.74 ± 0.04 ab	0.74 ± 0.03 bc
nZVI	T0	0.20 ± 0.01 cd	0.49 ± 0.01 h	0.59 ± 0.01 f	0.54 ± 0.01 h
	T1	0.21 ± 0.01 bc	0.52 ± 0.01 gh	0.61 ± 0.01 ef	0.59 ± 0.01 g
	T2	0.23 ± 0.01 ab	0.61 ± 0.01 de	0.64 ± 0.03 def	0.63 ± 0.01 fg
	T3	0.19 ± 0.02 cd	0.65 ± 0.01 cd	0.66 ± 0.02 cde	0.67 ± 0.01 ef
	T4	0.14 ± 0.01 e	0.69 ± 0.01 b	0.69 ± 0.01 bcd	0.78 ± 0.02 b
	T5	0.13 ± 0.02 e	0.56 ± 0.03 fg	0.71 ± 0.02 abc	0.72 ± 0.04 cd

Note: The fluorescence of Chinese cabbage under control, nZVI, and S−nZVI treatments. The 10−day−old seedlings were treated, and the leaves of 45−day−old seedlings were collected to measure the leaf fluorescence parameters (minimal fluorescence F0, maximum fluorescence Fm, PSII maximum photoelectrochemical quantum yield Fv/Fm, and actual photochemical efficiency of photosystem II). The mean value is ± SD (n = 6). Columns with different letter connections indicated a significant difference between treatments at the *p* < 0.05 level (Duncan’s test).

## Data Availability

The original contributions presented in this study are included in the article. Further inquiries can be directed to the corresponding author.
